# Natural Products for the Prevention and Treatment of Oral Mucositis—A Review

**DOI:** 10.3390/ijms23084385

**Published:** 2022-04-15

**Authors:** Ana Sofia Ferreira, Catarina Macedo, Ana Margarida Silva, Cristina Delerue-Matos, Paulo Costa, Francisca Rodrigues

**Affiliations:** 1 REQUIMTE/LAQV—Instituto Superior de Engenharia do Porto, Rua Dr. António Bernardino de Almeida 431, 4249-015 Porto, Portugal; asofiamferreira.95@gmail.com (A.S.F.); catpmacedo@gmail.com (C.M.); ana.silva@graq.isep.ipp.pt (A.M.S.); cmm@isep.ipp.pt (C.D.-M.); 2 UCIBIO—Applied Molecular Biosciences Unit, MedTech-Laboratory of Pharmaceutical Technology, Faculty of Pharmacy, University of Porto, 4050-313 Porto, Portugal; pccosta@ff.up.pt; 3 Associate Laboratory i4HB—Institute for Health and Bioeconomy, Faculty of Pharmacy, University of Porto, 4050-313 Porto, Portugal

**Keywords:** cancer, drug delivery, natural products, oral mucositis, treatment

## Abstract

Cancer, a major world public health problem, is associated with chemotherapy treatments whose administration leads to secondary concerns, such as oral mucositis (OM). The OM disorder is characterized by the presence of ulcers in the oral mucosa that cause pain, bleeding, and difficulty in ingesting fluids and solids, or speaking. Bioactive compounds from natural sources have arisen as an effective approach for OM. This review aims to summarize the new potential application of different natural products in the prevention and treatment of OM in comparison to conventional ones, also providing a deep insight into the most recent clinical studies. Natural products, such as *Aloe vera*, *Glycyrrhiza glabra*, *Camellia sinensis*, *Calendula officinalis*, or honeybee crops, constitute examples of sources of bioactive compounds with pharmacological interest due to their well-reported activities (e.g., antimicrobial, antiviral, anti-inflammatory, analgesic, or wound healing). These activities are associated with the bioactive compounds present in their matrix (such as flavonoids), which are associated with in vivo biological activities and minimal or absent toxicity. Finally, encapsulation has arisen as a future opportunity to preserve the chemical stability and the drug bioa vailability of bioactive compounds and, most importantly, to improve the buccal retention period and the therapeutic effects.

## 1. Introduction

Cancer is currently a major public health problem all over the world. In 2020, almost 19.3 millions new cases were diagnosed worldwide [1]. Treatment of malignancies with cytotoxic chemotherapy (CT), radiation (RT), or a combination of the two is becoming more effective, as it is associated with short- and long-term adverse effects, including mucositis [2,3]. This secondary reaction may occur in any area of the gastrointestinal tract’s mucosal layer, from the mouth to the anus, with the oral cavity being the most prevalent location. The cytotoxicity is caused by a variety of mechanisms, including inhibition of DNA replication and repair, cell-cycle arrest, DNA damage, and cell death [4]. However, the precise and complex molecular pathways underlying the oral epithelial damage are not completely known [5,6].

Oral mucositis (OM) is a painful inflammatory and frequently ulcerative disorder of the oral mucosa that severely reduces the patient’s quality of life [3,7,8]. OM occurs in 20 to 40% of the patients submitted to conventional CT, 80% of patients on high-dose CT, 75 to 100% of patients receiving hematopoietic cell transplants, and practically all patients with head and neck squamous carcinoma (HNSC) undergoing RT [4,5,9,10,11]. Common symptoms of OM include pain, bleeding, ulcers, and difficulty ingesting fluids or solids and speaking, as well as severe complications, such as secondary infections and significant weight loss, which may compromise the treatment of the primary disease and its outcome [2,12,13]. In addition, OM may result in the need for enteral or parenteral nutrition [14,15] and systemic analgesics [16,17,18], thus increasing hospitalizations [13,19], the use of resources and higher costs [19,20], and, in some cases, the risk of sepsis [8,21]. However, when mucositis progresses, topical analgesics become less effective and systemic opioids may be required [22,23,24]. Different strategies have been used to attempt the prevention or amelioration of this condition, and some clinical trials were effective [8,16,17,25]. For example, cryotherapy [11,26] and keratinocyte growth factor [11,27] demonstrated some benefits in preventing mucositis. Zinc [28,29] and vitamin E [28,30,31] were effective in reducing the severity of OM, but *Aloe vera* [32], amifostine [4,33], glutamine [28,30,34], honey [32,35,36,37,38], photobiomodulation (PBM) therapy [39,40,41], and antibiotics [21] demonstrated lower evidence of benefits. The studies reviewed were evaluated in patients with different types of cancer who underwent different treatment approaches.

While there are a growing number of innovative anticancer agents, few therapeutic alternatives for the prevention or treatment of oral mucositis have been reported. Most important, the scarce alternatives that have been successfully achieved are still unsatisfactory [6,16]. Therefore, the search for alternative compounds obtained from natural sources could be an option and a challenge for this research field. Natural compounds, in contrast to synthetic ones, are often thought to have fewer side effects, are easy to access, and present beneficial bioactive properties (e.g., anti-inflammatory, antioxidant, and antimicrobial properties), making them interesting solutions as promising therapeutics. Aside from the protective results of natural products against toxicity induced by radiation or antineoplastic drugs, one of the most promising preventive measures in patients during therapy may be the employment of natural products. The aim of this review is to provide an overview of the use of natural compounds for the prevention and eventual treatment of OM in cancer patients and their potential applications in drug delivery systems to overcome the specific limitations of the oral cavity environment.

## 2. Oral Mucositis

As previously stated, mucositis is an inflammatory response condition of the oral mucous membrane that is frequently observed in malignant neoplastic patients undergoing CT, RT, or both. This condition develops due to interactions among an oral tissue injury, the oral cavity environment, bone marrow suppression, and innate predisposing factors in the patient [18,42,43]. The symptoms of OM, such as oral mucosal atrophy, swelling, erythema and subsequent pain, bleeding, ulceration, difficulty in feeding and even swallowing saliva, or a combination thereof, may be diverse [2,11,44]. Difficulties with eating reduce the nutritional intake, resulting in a decline in the patient’s nutritional status. This can also seriously affect their speech due to an uncomfortably dry mouth and a decrease or increase in salivation [11,16,44]. OM may also be aggravated by injuries induced by sharpened teeth, bruxism, food, and microorganisms [44,45]. Naturally, additional ulcers provide an easy access point for microorganisms, including bacteria, fungi, and viruses, to enter the bloodstream because of the loss of mucosal integrity, culminating in systemic infections that may cause the treatment for fighting the primary disease to be discontinued or even threaten the patient’s survival. Moreover, the dysphagia, xerostomia, and changes in taste caused by OM can increase the systemic symptoms, such as lethargy and anorexia, as well as psychological issues. Consequently, OM is associated with increased resource needs and potentially major economic impacts—depending on its severity—due to the more frequent and prolonged hospitalizations for support and nutritional care and analgesic treatments.

Three tools are available for assessing the severity of OM. The most extensively used is the World Health Organization’s Oral Mucositis Grading Scale (WHO-OMGS), which incorporates clinical criteria to evaluate the OM lesion and eating capacity [46]. On the other hand, the Common Terminology Criteria for Adverse Events in its fifth revision (CTCAE v5.0) considers the following factors when assessing the impact of OM: pain intensity, ability to eat, and need for intervention [46]. Finally, Radiation Therapy Oncology Group (RTOG) defines the severity of RT-induced OM using a four-point scale [46]. OM is classified according to these three criteria, as summarized in Table 1.

### 2.1. Physiopathology of OM

**Figure 1:**
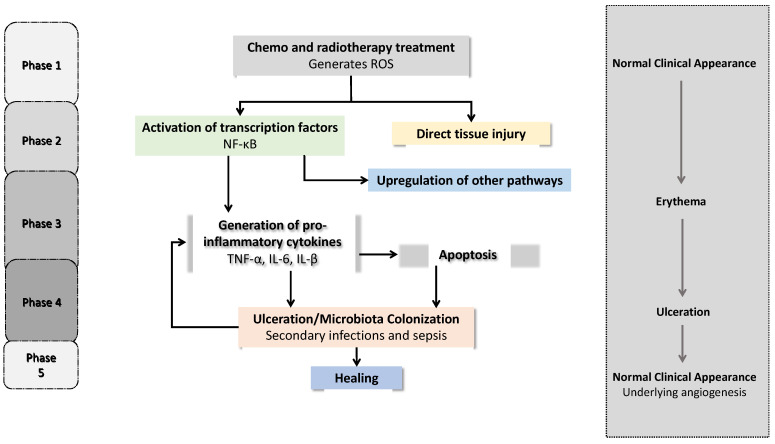
Diagram representing the mucosal cells and clinical manifestations of oral mucositis.

In the last decades, substantial evolution has taken place in the understanding of the complex mechanism behind the development of mucositis [6]. A five-phase model that begins with an (i) initiation involving cell injury, (ii) elevation of inflammatory cytokines, a (iii) primary damage response, and (iv) signaling and amplification of the inflammatory cascade, followed by (v) ulceration and mucosal repair through epithelial proliferation, has been reported by different authors [2,3,16,47]. Thus, OM is characterized by a cascade of events that occur simultaneously and are mechanistically related (Figure 1). Therefore, each factor that drives each phase may constitute a possible therapeutic target [16].

The mucositis initiation phase—initiation—corresponds to the injury of oral mucosal cells caused by CT and/or RT. This phase begins instantaneously as the antineoplastic treatment is being administered [5,6,48,49]. The second phase—upregulation with messenger generation—involves the cytotoxic effect, resulting in the generation of reactive oxygen and nitrogen species (ROS and RNS, respectively) and DNA damage, leading to basal and suprabasal epithelial cell death [2,3,6]. Particularly, when DNA strands breaks, the apoptotic process is activated, with p53 and nuclear factor κB (NF- κB) playing major roles [50,51]. At this point, inflammatory cytokines, chemokines, and adhesion molecules are generated when NF- κB, the key mediator of pro-inflammatory gene expression, is activated, which is clinically manifested as mucosal damage. The release of pro-inflammatory cytokines, such as tumor necrosis factor (TNF-α), interleukin-1β (IL-1β), and interleukin-6 (IL-6), is mediated through transcription factor activation, and this promotes connective tissue and endothelial damage, limiting the tissue oxygenation and stimulating epithelial basal cell death [2,18,50,51,52,53]. The third phase—signaling and amplification—is the consequence of tissue damage, apoptosis, enzyme activation, and vascular permeability, which amplify the molecules of the innate immune response as pro-inflammatory cytokines in a positive feedback mechanism, leading to more tissue damage [18,54]. In the fourth phase—ulceration—clinical signs of mucositis become visible, as the integrity of the mucosa and submucosa is disrupted, causing pain control to be required [3,5,16]. In neutropenic patients, the immune cells cannot respond properly, and the ulcerative lesions allow several microorganisms to penetrate into the connective tissue, triggering the production of more pro-inflammatory cytokines and increasing the tissue damage [50,51]. Bacteremia and sepsis are mostly caused by herpes simplex virus, *Candida albicans*, or other fungal genera, such as *Aspergillus* [10]. Healing usually occurs naturally after the cancer treatment is ceased, and it is marked by epithelial proliferation, migration, and differentiation promoted by the extracellular matrix [2,3,49]. The oral mucosa recovers, but the patient remains at risk for recurrent episodes due to residual angiogenesis [16,18,49,55].

CT patients often experience acute symptoms 3–5 days following its administration, with ulcerative lesions appearing a few days later and resolving within 2 weeks [3,44,51]. On the other hand, RT mucositis is a chronic condition that lasts up to 7 weeks. The radiation doses range from 2 to 70 Gy per day and cause ulcerations that remain for 3–4 weeks after the treatment is ceased [9,11,18]. The lack of taste develops because the oral mucosa is exposed to radiation after few weeks, compromising nutrition and psychological status, while the recovery begins 6–8 weeks after the completion of the treatment [5,9].

### 2.2. Risk Factors

The risk factors of OM can be classified as patient-related, tumor-related, and treatment-related variables, as summarized in Table 2 [16,45].

In the patient-related factors, gender has been linked to mucositis, since women are associated with a higher risk, which could be due to dosimetric considerations [12,25,58,62]. However, other studies reported the absence of evidence that gender and OM are correlated [16,45,58,63]. Although age is frequently reported as a mucositis risk factor, there are few consistent reports that link younger and older patients and mucositis severity [45,58]. Likewise, the effect of body mass index (BMI) on mucositis risk is inconsistent, with data suggesting that a low BMI and a BMI higher than 25 are related with a superior risk, as body composition can affect drug metabolism, as can smoking and poor oral hygiene [18,45,58]. Genetic variants, previous treatment, and comorbidities (such as renal dysfunction and diabetes mellitus) have been indicated as possible factors for chronic OM associated with RT [16,61].

In what concerns the tumor’s nature, its location, size, and stadium may also influence the grade of OM [45]. For instance, in HNSC patients, the standard protocol includes RT with a specific area and prescription dose, which influences the exposure to radiation and the subsequent mucosal damage [16,45]. However, in recent years, there was an increased investment in intraoral medical devices that enable the minimization of excessive irradiation of normal tissues [64].

Although the risk factors of OM are not completely understood, the characteristics of anticancer therapeutics (mechanism of action, dose, planning, and number of cycles) are closely associated with the prevalence and severity of the lesions, as their effects accumulate [12,18,45]. It is well known that female patients using methotrexate and melphalan have a greater chance of developing this local inflammatory condition [16].

Along with investigating intrinsic patient characteristics, such as pre-existing medical conditions, altered oral dynamics, and general health, age, oral health (hygiene prior to treatment), nutritional status, and liver and kidney function are critical, as they are parameters that a medical team must consider [2,18]. Aside from that, it is necessary to emphasize that OM is frequently documented only in its advanced phases owing to the requirements for clinical therapy and assistance [13,14,19,65]. Therefore, the search for new active ingredients that could be used in the prevention (and even treatment) of OM is of utmost importance.

### 2.3. Prevention and Management of OM

OM management strategies include either preventive or symptom control strategies [8,18,23,25]. The primary key measure in preventing OM is the preservation of tissue during RT treatment planning and the use of RT procedures that conserve the uninvolved oral mucosal surface [17,66]. Some strategies are addressed in the evidence-based guidelines developed by the Multinational Association of Supportive Care in Cancer and the International Society of Oral Oncology (MASCC/ISOO), which present three categories: a recommendation, a suggestion, and a situation where no guideline is possible [54,63]. These guidelines can be adjusted at any time to compensate for possible restraints in the clinic and patient choices [63]. Table 3 summarizes the recommended or suggested strategies for most of the groups of cancer patients.

Proper oral health and hygiene are essential for mitigating the risk and severity of OM [11,60]. Before initiating CT or RT, all potential causes of mucosal irritation should be removed, as they may worsen and prolong the development of oral mucositis [60]. Teeth with sharp surfaces must be restored, orthodontics and protheses should be removed, and the maintenance of a stable oral microbiome is also an important aspect. The presence of a balanced nutrition is another variable that may help in the relief of discomfort from mucositis [9,11,60]. Alcohol, smoking, and foods that are crunchy, acidic, spicy, or sweetened should be limited or eliminated [81].

As previously stated, OM can make the ingestion process a challenge, as it is normally unpleasant and, in extreme cases, impossible due to painful symptoms; therefore, a liquid diet is the only solution [2,18]. Therefore, soft and liquid diets may be necessary, and, in the case of patients that cannot tolerate a liquid diet, the solution is parenteral nutrition [17]. The patients’ complaints can be reduced with the use of specific mouthwashes with topical analgesics, anesthetics, antibiotics, and steroids [67,74,80,82], as topical analgesics and anesthetics are intended to relieve localized pain [23].

According to the MASCC/ISOO guidelines (Table 3), a benzydamine mouthwash may be useful due to its anti-inflammatory properties, which inhibit the production of TNF-α and IL-1β [46,53,63,74]. However, the use of saline, sodium bicarbonate, and antimicrobial (e.g., chlorhexidine 0.12%) rinses can ameliorate the symptoms of moderate mucositis [53,83]. In clinical practice, topical analgesics (e.g., morphine, benzocaine, and menthol) are applied to provide temporary relief in some patients, but their concentrations are not well established [22,84].

Currently, palifermin was the only agent that has been approved by the European Medical Agency (EMA) and the American Food and Drug Administration (FDA) for the prevention of OM in HSCT patients receiving CT and RT. However, on 1 April 2016, the European Commission withdrew the marketing authorization for this drug in the European Union (EU). The withdrawal was at the request of the marketing authorisation holder, which notified the European Commission of its decision to permanently discontinue the marketing of the product for commercial reasons. It has also been tested in HNSC patients in terms of its reduction of the state of pathogenic severity [10]. The MASSC/ISOO guidelines also indicate cryotherapy and photobiomodulation (PBM) protocols for more advanced phases. In particular, cryotherapy has been reported to reduce the symptoms of oral mucositis in patients undergoing CT as a result of its vasoconstriction, decrease in the blood flow, and reduction of the local distribution of the chemotherapeutic agent (e.g., fluorouracil (5-FU) and melphalan) [23,71]. Thirty minutes of ice chips used prior to the administration CT are the recommended and tolerable period [71,73].

PBM is another method employed to stabilize and inhibit the development of OM [39,85]. It has anti-inflammatory effects, diminishes the pain, and improves the healing rate of the basal wound. The energy applied to the specific area must be adapted according to the patient’s lesion. To relieve the most common complaints, PBM can be used both prophylactically and therapeutically, that is, it can be used before and after an antineoplastic treatment [40,86,87]. Mucositis may also be treated with supplementary vitamins and minerals. For instance, vitamin E, a potent antioxidant, may reduce the grade of mucositis by preventing the damage caused by ROS [2]. A blood test performed in severe OM patients demonstrated a lack of some vitamins (such as vitamins E, A, and D), which inhibited the pro-inflammatory pathways [34]. Different studies also showed that oral zinc supplements may be applied as a prophylactic treatment [18,29,30].

Therefore, for most of the strategies recommended or suggested in Table 3, the research in the literature displays minimal evidence or even contradictory results, thus invalidating the definitions of the guidelines [7,8,54,63]. Consequently, the search for new active ingredients with potential therapeutic effects for preventing or treating OM is a challenge. Natural compounds, the majority of which are rich in polyphenols, are an option that should be explored.

## 3. Natural Compounds and Their Properties for Preventing/Treating OM

Currently, the protocols and therapeutical agents available from the different authorities have the purpose of ameliorating the OM grade, as mentioned in the previous section, but no treatments with reasonable results have been established [42,53,88]. Aside from that, many of these compounds have been associated with adverse effects and high costs [8,19]. Thus, natural products, such as honey, *Aloe vera*, curcumin, or propolis, are of huge interest for the nutraceutical and pharmaceutical industries, as they are easily accessible and allow more cost-effective treatments with minimal or no toxicity when compared to conventional strategies [89,90]. Their richness in bioactive compounds with anti-inflammatory, antioxidant, antiseptic, analgesic, and wound-healing properties that may interfere with many cellular signaling pathways could play an important role in the progression of OM and the activity of carcinogenic cells (e.g., HNSC) [10].

### 3.1. Bee Products

Honey is a natural product generated by bees and has been used since ancient times in traditional medicine. The huge diversity of studies has shown the multiplicity of beneficial applications of honey based on its antioxidant, anti-inflammatory, antibacterial, antiviral, antifungal, antitumoral, antimutagenic, and wound-healing properties [32,35,37,38,77]. The composition of honey is difficult to exactly define, as the components and relative amounts are conditioned by the flora of the geographical area from which honeybees collect pollen [91]. In a general way, honey is a heterogeneous mixture of water, nectar sugars, and glandular secretions produced by honeybees that contain proteins, vitamins, and enzymes [92]. One of the enzymes present is glucose oxidase, which, when in contact with body tissue, may stimulate the production of hydrogen peroxide, which acts as a messenger and promote wound healing and rapid epithelization at low concentrations by stimulating the proliferation of fibroblasts and epithelial cells [92,93]. It is also suggested that matrix metalloproteases of connective tissue and neutrophil serine proteases may be activated by hydrogen peroxide [94]. Furthermore, the levulose and fructose present in honey may improve local nutrition and promote epithelialization [93,94]. Honey also has immunomodulatory effects, as it influences the activation of macrophages and the proliferation of B-lymphocytes and T-lymphocytes [95], in addition to decreasing the inflammatory process by inhibiting cyclooxygenase pathway and reducing prostaglandin synthesis [96]. The beneficial effects of honey may also be due to its moisturizing effect, low pH, and viscosity which inhibit the proliferation of bacteria [35].

Charalambous et al. conducted a randomized, controlled trial to evaluate the potential effect of thyme honey rinses on HNSC patients [35]. In this study that involved 72 participants, a solution of 20 mL of thyme honey diluted in 100 mL of purified water was given to the patients to gargle in the oral cavity three times per day (15 min before and after the RT session and 6 h later) for 7 weeks, starting from the first day of the fourth week of RT. The results showed a significant improvement (*p* < 0.001) in the patients’ quality of life, leading to fewer symptoms and maintenance of the body weight (*p* = 0.001) when compared to saline rinses [35]. Honey mouthwash also proved to be effective in a randomized, single-blind controlled trial that enrolled 53 patients [97]. The honey solution (honey-to-water ratio of 1:20) at 37 °C was gargled and kept in the mouth before and after each meal and before sleeping for 30 s by the treatment group, while the patients in the control group received routine care, such as ingestion of fluconazole capsules, nursing care, and mouth hygiene training [97]. According to the authors, the solution reduced or eliminated weight loss, leading to some weight gain and preventing and reducing the severity of OM in the acute myeloid leukemia patients receiving CT (*p* < 0.001 at the fourth week of treatment) [97]. In another randomized, controlled trial with a parallel design involving 150 children, Sener et al. treated 25 OM patients with honey (with vitamin E as the most effective compound) for 21 days, applying the amount of 1–1.5 g of honey per weight (kg) of the child twice per day (every 12 h) [31]. Honey was found to be more effective in the management of OM (*p* < 0.05) when compared to chlorhexidine, a wide-spectrum antifungal and bactericidal antiseptic solution that is frequently used in oral care [31]. Motallbnejad et al. also conducted a randomized single-blind (examiner-blind) clinical trial to evaluate the effect of pure honey on radiation-induced mucositis in a total of 40 patients with head and neck cancer receiving RT [95]. Twenty patients were instructed to rinse and gradually swallow 20 mL of pure honey 15 min before radiation, then again at intervals of 15 min and six hours after radiation, while the control group was advised to rinse with 20 mL of saline before and after radiation. This procedure was repeated weekly from the beginning of the treatment until the end of the RT. The honey-receiving patients exhibited a significant reduction in OM (*p* < 0.001) when compared to the control group [95]. In a unicenter randomized, controlled clinical human study involving 82 patients with head and neck cancer treated with RT over 4–6 weeks, the treatment group was instructed to take 20 mL of Ziziphus honey 15 min before and after the radiation and before sleeping at night, while the control group repeated the process using 20 mL of 0.9% saline [92]. The results showed that the proportion of mucositis (Grades 3 and 4) was lower in the honey-treated group (*p* = 0.016 and *p* = 0.032 for Grades 3 and 4 of mucositis, respectively) than in the control group at the end of 6 weeks of RT [92]. In 2010, Khanal et al. conducted a single-blinded, randomized, controlled clinical trial over 6 weeks on 40 oral carcinoma patients receiving RT [91]. Radiation was given once per day for 5 days a week, and the application was performed 15 min before and after radiation and once before going to bed. Honey extracted from beehives of the Western Ghats forests or lignocaine gel 2% (control group) was swished around the oral cavity for 2 min and expectorated. Only one of the 20 patients of the treatment group developed intolerable mucositis (*p* < 0.0001) compared to 15 of the 20 patients of the lignocaine group [91]. Caffeine, a natural alkaloid with hypoalgesic, antioxidant, and anti-inflammatory effects, has also been screened as a potential ingredient to work against oral mucositis [98,99,100,101]. In a double-blinded randomized clinical trial involving 75 patients (that randomly fell into three treatment groups) presenting OM after CT, the therapeutic effects of coffee plus honey were compared with those of topical steroids that are usually used in the treatment of OM after CT [98]. A syrup-like solution was prepared for each treatment group: 300 g of honey plus 20 g of instant coffee for the honey-plus-coffee group; 300 mg of honey for the honey group; the control group was treated with 20 eight-milligram ampoules of betamethasone solution. All groups were instructed to sip 10 mL of the prescribed product and swallow every 3 h for 1 week. While all treatment regimens decreased the severity of the lesions, the best result was achieved in the honey–coffee group (*p* < 0.05), followed by the honey-and-steroid groups [98].

#### 3.1.1. Propolis

Propolis is a resinous material produced by bees and is frequently used as natural nutritional supplement [102]. It is composed of a mix of plant buds and exudates, bee enzymes, pollen, and wax, and it has been widely used by different civilizations to treat colds, wounds, and ulcers due to its anesthetic, antimicrobial, anti-inflammatory, antitumor, immunomodulatory, and antioxidant properties [102]. Similarly to honey, the chemical composition of propolis is highly dependent on the diversity of the flora and bee species [103,104]. It is mainly composed of proteins, amino acids, vitamins (A, B1, B2, B3, and B7), minerals, essential oils, phenolic acids, alcohols, fatty acids, and flavonoids [102,105,106,107,108]. Regarding OM, the bioactivity of propolis is mainly associated with flavonoids, as these molecules are capable of sequestering or inhibiting the formation of free radicals, and they promote immunomodulatory, antioxidant, wound-healing, and anti-inflammatory activities [109]. The anti-inflammatory properties are directly related with the inhibition of the synthesis of prostaglandins and promotion of phagocytic activity [110]. In addition, propolis promotes healing effects in epithelial tissues, while the presence of iron and zinc improves the synthesis of collagen [108].

Akhavan-Karbassi et al. conducted a randomized double-blind placebo-controlled trial to evaluate the potential effect of propolis mouthwash on head and neck tumor patients undergoing CT [111]. In the treatment group (*n* = 20), 5 mL of propolis mouth rinse (30% extract) was administered every 8 h for 7 consecutive days. The solution was swished in the patients’ mouths for 60 s, gargled, and expectorated. In the control group (*n* = 20), the process was repeated with a placebo mouth rinse. OM, erythema, and eating and drink ability were evaluated. When compared to the control group, the treatment group presented significant improvement in OM, wound healing, and erythema at day 7 (*p* = 0.006), but no significant differences in eating and drinking ability were observed (*p* = 0.21). Moreover, 65% of the patients in the propolis group were completely healed by day 7 of the trial [111]. 

#### 3.1.2. Royal Jelly

Royal jelly is a secretory product of the cephalic glands of nurse bees that serves as the diet of honeybee larvae in their first 2–3 days, while for the queen, it is the specific food for her whole life period [112]. It is widely used in folk and mainstream medicines and as a dietary supplement due to its antioxidant, anti-inflammatory, hypoglycemic, antibiotic, antitumor, antiallergic, antiaging, immunomodulatory, neurotrophic, hypocholesterolemic, hepatoprotective, hypotensive, and blood pressure regulatory activities [112,113,114,115,116,117,118,119,120].

Similarly to the aforementioned bee products, the composition of royal jelly is dependent on the geography and climate [121]. It is a complex substance with a unique combination of sugars (mainly glucose and fructose, as well as traces of sucrose, maltose, trehalose, melibiose, ribose, and erlose), proteins (which represent >50% of the dry weight of royal jelly), amino acids, nucleotides, ascorbic acid, phenols, waxes, fatty acids, steroids, and phospholipids [121]. The impact that royal jelly has on OM may be closely related to its anti-inflammatory and wound-healing activities. However, the active compounds of royal jelly and the mechanisms underlying these activities are still largely unknown. In vitro studies performed on mice revealed that supernatants of royal jelly suspensions added to a mouse peritoneal macrophage culture stimulated with lipopolysaccharides and IFN-γ efficiently suppressed the secretion of pro-inflammatory cytokines TNF-α, IL-6, and IL-1, which was probably due to protein factors such as Major Royal Jelly Protein 3 (MRJP3) [119]. MRJP2, MRJP3, and MRJP7 are thought to be responsible for the wound-healing bioactivity of royal jelly, as they stimulate cell migration and proliferation [122], along with the antioxidant compounds present in royal jelly, which, when taken orally, lowered the levels of 8-hydroxy-2-deoxyguanosine, a marker of oxidative stress in mouse kidney DNA and serum [123].

Suemaru et al. evaluated the effects of royal jelly, honey, and propolis on OM induced with 5-fluorouracil and mild abrasions made on the cheek pouch in hamsters [124]. The bee products were topically administered to the oral mucosa. Royal jelly ointments at 3%, 10%, and 30% improved the recovery from 5-fluorouracil-induced damage in a dose-dependent manner, while the results of ointments of honey at 1%, 10%, and 100% and propolis at 0.3%, 1%, and 3% were not statically different from those of the Vaseline-treated control group [124]. In a more in-depth trial in Golden Syrian hamsters, the influence of royal jelly on 5-fluorouracil-induced OM was assessed using oral mucosal adhesive films containing royal jelly [125]. The 5-fluorouracil was administered through intraperitoneal injections on days 0 and 2, and the left cheek pouches of hamsters (*n* = 12 per group) were everted and scratched with a small wire brush on days 1 and 2. Royal-jelly-containing sodium alginate–chitosan films (10% or 30%) were applied to the cheek pouches every day from day 3. Royal-jelly-containing films (both 10% and 30%) improved the recovery from 5-fluorouracil-induced OM, which presented lower erythema and absence of ulceration and abscesses on day 8. They also reduced the myelo-peroxidase (MPO) activity and the expression of pro-inflammatory cytokines. The data suggest that these effects were caused by the anti-inflammatory or antioxidative properties of royal jelly [125]. In humans, the effect of royal jelly on OM in patients with different types of malignancies undergoing RT and CT was evaluated by Erdem et al. in a randomized, controlled trial [126]. In this clinical trial that involved 103 patients, all patients received a mouthwash therapy with benzydamine hydrochloride and nystatin rinses. In addition, patients in the experimental group received royal jelly two times per day for a total daily dose of 1 g. Royal jelly was orally swished for 30 s and swallowed. The treatment group showed a mean resolution time of OM that was significantly shorter than that of the control group (OM Grade 1: *p* = 0.0001; OM Grade 2: *p* = 0.0001; OM Grade 3: *p* = 0.05) [126]. In a single-blind clinical trial that involved 13 patients with head and neck cancer receiving CT, 1 g of royal jelly was given three times per day to the treatment group during the RT period [127]. Royal jelly was shown to have a preventive effect on the progression of CT-induced OM from the early phase (*p* < 0.001) [127].

### 3.2. Spondias Mombin

The leaves of *Spondias mombin*, commonly known as the cashew tree, are a rich source of interesting bioactive compounds, with particular emphasis on tannins, saponins, triterpenes, and flavonoids [128]. Traditionally, the leaves have been used to treat inflammatory pathologies, making them a promising source for the development of new therapeutic agents for OM [128]. Gomes et al. assessed the effects of a hydroethanolic extract of *S. mombin* leaves on 5-fluorouracil-induced OM in Golden Syrian male hamsters [128]. The animals were orally pre-treated with the hydroethanolic extract of *S. mombin* leaves (50, 100, or 200 mg/kg) for ten days [128]. The treatment with the highest dose of the extract (200 mg/kg) showed the best healing effect, with hamsters displaying reduced oxidative stress and inflammation and no evidence of ulceration. Further analysis showed re-epithelialization, absence of hemorrhage, discrete mononuclear inflammatory infiltration, and lower expression levels of different molecules involved in the modulation of inflammation, such as MMP-2, COX-2, TNF-α, NF-κB p50 NLS, iNOS, and IL-1β, as well as an increase in glutathione (GSH) levels [128]. Although the mechanisms behind these effects remain under investigation, the hydroethanolic extract of *S. mombin* leaves is rich in potent antioxidant phenolic phytochemicals, such as ellagic acid (12 mg/g) and chlorogenic acid (19.4 mg/g), which could justify these activities [129]. Studies have demonstrated that chlorogenic acid acts on the reduction of COX-2 expression in macrophages, as well as in the inhibition of the production of pro-inflammatory cytokines, such as IL-1β and TNF-α, and of NF-κB activation [129]. On the other hand, chlorogenic acid was proven to promote wound healing in rats [130], while ellagic acid acted by down-regulating MMP-2 expression and inhibiting NF-κB-mediated transcriptional activation [129]. These activities may justify the results achieved in the previously detailed trial.

### 3.3. Camellia sinensis

*Camellia sinensis* (green tea) is one of the most popular drinks in the world and is widely known for its antimicrobial, antitumoral, antioxidant, and anti-inflammatory activities [67]. Different compounds with therapeutic effects have been discovered in this plant. The majority of the health-promoting properties are associated with polyphenols [131], which represent almost 30% of the fresh-leaf dry weight, including flavandiols, flavonols, flavonoids, and phenolic acids [132]. However, most of the polyphenols present in leaves of *C. sinensis* are catechins, namely, (+)-catechin, (−)-epicatechin, (+)-gallocatechin, (−)-epigallocatechin (EGC), (−)-epicatechin gallate, and (−)-epigallocatechin gallate (EGCG) [133]. Catechins are mainly responsible for the ROS scavenging and antioxidant activities of *C. sinensis* [134,135]. EGCG, in particular, efficiently inhibits the transcription of NF-κB, resulting in a decrease in the expression of different pro-inflammatory genes [136]. The anti-inflammatory effect of catechins may be due to the activation of endothelial nitric oxide synthase (eNOS) [137,138].

The effect of green tea on OM was evaluated in oral cancer patients [67]. For that, a single-blind randomized, controlled trial was made with 63 participants. For 6 months, after the tooth-brushing procedure, the intervention group rinsed the mouth with a solution of 5 g of green tea dissolved in 100 mL of water for 60 s, and the control group rinsed the mouth with 100 mL of tap water for the same period. The results demonstrated an improvement in oral health status and the preservation of the oral mucosa at the end of the follow-up period (6 months), with a higher reduction of the oral health status score in the intervention group than in the control group (*p* = 0.008) [67]. In another randomized study, the effect of Baxidil Onco^®^ mouthwash (Sanitas Farmaceutici Srl, Tortona, Italy), composed of *C. Sinensis* leaf extract and palmitoyil hydrolyzed wheat protein, was tested in 60 hematologic patients undergoing hematopoietic stem cell transplantation (HCST) [139]. Twenty mL of Baxidil Onco^®^ was used to rinse the mouths of 28 patients four times per day for at least one minute without swallowing, while the remaining 32 patients were treated with standard prophylactic schedules and served as control. The results demonstrated that the incidence, severity, and duration of OM were significantly reduced (*p* = 0.022) by the oral rinsing with Baxidil Onco^®^ [139].

### 3.4. Plantago Major

In traditional Persian medicine, *Plantago major* was used as a wound-healing herb, as it possesses a wide range of bioactive properties, such as anti-inflammatory, antiulcerogenic, antioxidant, antimicrobial, analgesic, wound-healing, and immunomodulatory effects [140].

Soltani et al. conducted a randomized, double-blind, placebo-controlled clinical trial to assess the effects of *P. major* syrup as a natural agent against OM for 7 weeks [141]. The participants were HNSC patients who were going to receive RT. The 23 patients of the intervention group received 7.5 cc of *P. major* syrup three times per day, starting from three days before the start of RT until the end of it, while the placebo group received 7.5 cc of placebo syrup. The *P. major* syrup was shown to be effective in the reduction of the mucositis and the severity of pain caused by RT (*p* < 0.001) [141]. A multicenter randomized, controlled trial developed by Cabrera-Jaime et al. evaluated the efficacy of *P. major* extract vs. chlorhexidine vs. sodium bicarbonate in the treatment of CT-induced OM in solid-tumor cancer patients with grade II–III mucositis [140]. A total of 45 patients were randomized for one of the treatments, consisting of a 5% aqueous solution of sodium bicarbonate together with (i) an additional dose of 5% sodium bicarbonate, (ii) *P. major extract*, or (iii) 0.12% chlorhexidine. The solutions were applied over 14 days. The differences in healing time and the lower pain levels among the three groups were not statistically significant (*p* = 0.702) [140].

The properties of *P. major* leaves are dependent on the different compounds present. The leaves are rich in different bioactive molecules, such as aucubin, a glycoside with anti-toxin activity, and ursolic, oleanolic, and α-linoleic acids, which inhibit COX-2-catalyzed prostaglandin production [142,143,144]. Extracts of *P. major* leaves have remarkable antioxidant and antiradical capacities due to the presence of baicalein, lutolin, salicylic acid, citric acid, ascorbic acid, apigenin, ferulic acid, benzoic acid, chlorogenic acid, oleanolic acid, and ursolic acids [145,146]. According to different studies, the bioactivity of *P. major* is due to the decrease in the inflammatory reaction through the modulation of NF-κB, NO, COX-2, and B4 leukotriene (LB4) levels [140].

### 3.5. Aloe vera

*A. vera* is a plant that has been used for medical purposes for thousands of years. It is widely employed for the treatment of various medical conditions, such as oral ulcers, psoriasis, skin burns, and frostbite, since it presents analgesic, liver-protection, antifungal, antidiabetic, anti-inflammatory, antiproliferative, anticarcinogenic, antiaging, and immunomodulatory properties [147,148,149]. In addition, it can scavenge free radicals, improve wound oxygenation, promote wound healing, increase collagen formation, and inhibit metalloproteinase and collagenase activity [150,151,152,153,154]. Different studies have shown the potent free-radical and superoxide anion activity of three derivatives from *A. vera*, namely, isorabaichromone, feruoylaloesin, and *p*-coumaroylaloesin [150,151]. The beneficial effects, assumed to be exerted in the oral cavity, may also be due to its moisturizing effect, which is provided by the polysaccharide components (principally mannose, glucose, xylose, arabinose, galactose, and rhamnose), which provide and sustain moisture in tissues [155]. One of the sugars present in a higher quantity, mannose-6-phosphate, acted as an active-growth substance and anti-inflammatory agent in in vivo studies on mice [156]. The anti-inflammatory effects of *A. vera* extracts are attributable to the inhibitory action on the arachidonic acid pathway via COX-2 inhibition [150,157], as well as the reduction of leukocyte adhesion molecules and TNF-α levels [158]. In vitro and animal assays suggest that *A. vera* promotes wound healing through the reduction of the vasoconstriction and the platelet aggregation at the wound site [152].

An initial assessment of *A. vera*’s potential in preventing RT-induced OM did not yield promising results in a single-institution, double-blind, prospective, randomized trial that involved 58 head and neck cancer patients [159]. The patients were instructed to take a 20 mL swish (*A. vera* solution or placebo) and swallow four times daily, beginning on the first day and continuing throughout the course of RT. However, no significant differences were observed between treatments (*p* = 0.07) [159]. Better results were achieved in other studies. Mansouri et al. evaluated the effect of *A. vera* on CT-induced OM in patients with acute lymphocytic leukemia and acute myeloid leukemia [160]. In this randomized, controlled clinical trial, 64 patients were divided into an intervention group and a control group. The first group was instructed to wash their mouths with 5 mL of *A. vera* solution for 2 min three times per day for 14 days. The control group repeated the procedure using mouthwashes that are typically recommended by hematologic centers, including normal saline, nystatin, and chlorhexidine. An evaluation of the patients’ mouths was performed on days 1, 3, 5, 7, and 14. Even though, regarding the intensity of stomatitis and pain, no significant differences were found between the two groups on the first day, a significant difference was observed in this regard on the other days (*p* < 0.001) [160]. In a similar study, an assessment of the effect of *A. vera* mouthwash on CT-induced OM was performed in a double-blinded randomized clinical trial on 120 patients, who were divided into three groups [161]. Until 2 weeks after the CT sessions, group 1 received tablets with 10 mg of atorvastatin daily plus a placebo mouthwash, group 2 received placebo tablets and *A. vera* mouthwash, and group 3 received placebo tablets and placebo mouthwash. The analysis of the results showed that 50% of the placebo patients (group 3) experienced mucositis, while that value decreased to 2.5% in group 2 (*p* < 0.042), with no significant differences between groups 1 and 3 (*p* < 0.674) [161]. Likewise, the efficacy of *A. vera* use for prevention of CT-induced OM was evaluated in a randomized, controlled clinical trial in 26 children with acute lymphoblastic leukemia [162]. Depending on the treatment group, a 70% *A. vera* solution or a 5% sodium bicarbonate solution was applied twice per day to oral tissues with spongeous sticks. The application started 3 days before the CT therapy. The application of *A. vera* solution showed to be effective in the prevention and reduction of OM severity (*p* < 0.001) [162]. A triple-blind randomized and controlled interventional quality-of-life clinical trial on the efficacy of *A. vera* and a benzydamine mouthwash in the alleviation of RT-induced OM was performed by Sahebjamee et al. in a study with 26 head and neck cancer patients [163]. The intervention group rinsed the mouth three times per day with 5 mL of an *A. vera* mouthwash, while the control group repeated the procedure with benzydamine mouthwash. The protocol was applied from the first day of RT until the end of the treatment, demonstrating that *A. vera* mouthwash was as efficient as benzydamine at reducing the severity of RT-induced OM, without differences between them (*p* < 0.09) [163].

### 3.6. Curcuma Longa

*Curcuma longa*, also known as turmeric, is an herb that is extensively grown in Asia [164] and is often used culinarily as a spice and in traditional Asian medical treatments for depression, stress, infection, and dermatological diseases [165,166]. Various compounds were identified in this plant, including polyphenols, sesquiterpenes, diterpenes, triterpenoids, sterols, and alkaloids [165,167]. Among these, the most studied component of *C. longa* is curcumin, a lipophilic polyphenol extracted from the rhizomes of *C. longa*, which represent 2–5% of turmeric [164,165].

Due to the antioxidant, anti-inflammatory, and anticancer effects of curcumin, it has an important role in the prevention of depression, cancer, and pro-inflammatory, neurodegenerative, diabetic, autoimmune, and cardiovascular diseases [168,169,170,171,172]. Furthermore, curcumin has antimicrobial, insecticidal, larvicidal, and radioprotective activities [165]. Curcumin mediates its effects through direct or indirect interactions with growth factors, kinases, enzymes, transcription factors, receptors, and proteins that regulate cell proliferation and apoptosis [168,173,174,175]. In the case of OM, the beneficial effects of curcumin may be related with the upregulation of TGF-β-1, which promotes re-epithelialization through the stimulation of fibronectin and collagen production by fibroblasts, while increasing the rate of granulation [168,176,177]. TGF-β-1also promotes the removal of dead tissue by enhancing the recruitment of macrophages [177]. Aside from that, curcumin potently inhibits the activation of nuclear factor-κB (NF-κB), but activates others, such as the nuclear factor erythroid 2-related factor 2 (Nrf2) [168,176,177]. COX-2, the inducible form of COX, can be selectively induced by mitogenic and inflammatory stimuli, resulting in enhanced synthesis of prostaglandins, such as IL-6. The activation of NF-κB significantly upregulates superoxide dismutase (SOD) expression [168,176,177]. Curcumin also enhanced the expression of antioxidant enzymes such as SOD, catalase (CAT), glutathione (GSH), and glutathione peroxidase (GSH-px) through the regulation of Nrf2 [168,176,177].

The wound-healing ability of curcumin is accelerated by its antioxidant activity, as it decreases the levels of lipid peroxides (LPs) and increases the activity levels of superoxide dismutase (SOD), catalase (CAT), and glutathione peroxidase (GPx) [178].

In a placebo-controlled study, an assessment of the tolerability of a curcumin mouthwash for the prevention of OM in pediatric patients undergoing CT was performed in a group of seven pediatric and young-adult patients [179]. In this study, which was developed without a control group for ethical reasons, in addition to the standard preventive oral care consisting of 0.2% chlorhexidine mouthwash for 30 s twice per day, the patients also used a mouthwash with 10 drops of Curcumall^®^ (a dietary supplement containing turmeric, curcumin and ginger) twice per day during the CT treatment. The researcher concluded that curcumin mouthwash was safe and well tolerated by the patients [179]. The efficiency of curcumin mouthwash in cancer patients undergoing RT and suffering from OM was evaluated in a randomized trial involving 20 patients [180]. The study group used 0.004% curcumin mouthwash diluted at a ratio of 1:5 for 1 min three times per day for 20 days, while the control group was treated with standard preventive oral care using a commercially available 0.2% chlorhexidine mouthwash to be used in a 1:1 dilution for 1 min three times per day for 20 days. Curcumin promoted faster wound healing and better patient compliance in the management of RT-induced OM (*p* < 0.001) [180]. In another double-blind randomized clinical trial, the effects of curcumin encapsulated in nanomicelles on OM in 32 head and neck cancer patients receiving RT were evaluated [181]. During the RT, patients in the treatment group received daily one capsule of SinaCurcumin^®^ (Exir Nano Sina Company, Tehran, Iran), which contained 80 mg of curcumin-loaded nanomicelles. The control group received placebo tablets containing lactose. There were statistically significant differences (*p* < 0.05) between the two groups in the severity of OM, as all of the patients in the placebo group developed OM versus the 32% of the case group [181].

### 3.7. Olea Europaea

Olive leaf extract is a natural product extracted from *Olea europaea*, which is traditionally used to treat and prevent hypertension and diabetes due to its antioxidant, anti-inflammatory, anticancer, antiapoptotic, antimicrobial, hypoglycemic, and diuretic properties [182,183,184,185,186,187]. The leaves of *O. europaea* contain a high concentration of phenolic compounds (1450 mg/100 g of fresh leaf), with secoiridoid oleuropein, verbascoside, rutin, luteolin-7-glucoside, and hydroxytyrosol as the main phenolic constituents [188]. Oleuropein is possibly the main active compound promoting the wound-healing activity of olive leaf extract, as it increases collagen fiber deposition and advanced re-epithelialization [189,190]. Furthermore, it has been demonstrated that oleuropein decreases oxidative stress and inflammation through the modulation of the COX-2, AMPF, eNOS, MAPK, and apoptosis cell signaling pathways in in vivo studies on mice [187]. In addition, olive leaf extract also inhibited the aggregation platelets in in vitro studies [186].

In 2013, the effect of a mouth rinse containing olive leaf extract on the prevention of severe OM in CT-receiving patients, as well as an estimation of its effect on the salivary levels of pro-inflammatory cytokines, was assessed in a prospective, randomized, double-blind, placebo-controlled cross-over study design involving 25 cancer patients [182]. The studied drugs (olive leaf extract at 333 mg/mL, benzydamine hydrochloride at 0.15 g/100 mL, or normal saline) were self-administered 3–4 times daily for 14 days, starting on the first day of chemotherapy. The patients were evaluated weekly until 15 days after CT for each cycle. The findings indicated that the olive leaf extract could effectively reduce the OM rates (*p* < 0.001) by decreasing the salivary levels of IL-1β and TNF-α [182]. Briefly, Ahmed et al. performed an experimental animal study and a prospective, randomized, double-blind, placebo-controlled cross-over study to evaluate the management of OM with mouthwashes containing olive leaf extract [191]. In the animal study, 45 male albino rats received two intraperitoneal injections of 5-fluorouracil (60 mg/kg) on day 0 and day 2. The first group received normal saline, the second group received olive leaf extract (333 mg/mL), and the third group received benzydamine hydrochloride (0.15 g/100 mL). By the end of the study (day 14), the control group presented ulcerated connective tissue that was not completely covered by epithelium, and there was evidence of necrosis and degeneration. The animals with the olive leaf extract and benzydamine hydrochloride presented a totally re-epithelialized mucosal surface with hyperkeratinization and hyperplasia, while the sub-epithelia were more organized, with decreased cellularity of fibrous tissue [191]. In a clinical study, 62 CT-receiving patients were divided to receive olive leaf extract, benzydamine hydrochloride, or a placebo in the form of a mouth rinse, and the treatment was changed in the next chemotherapy cycle for each patient (cross-over design) [191]. Mouth rinses were self-administered 3–4 times per day for 14 days from the start of the CT. When compared to the benzydamine hydrochloride and the control, the olive leaf extract more efficiently reduced the oral pain, dysphagia, and functional impairment of eating (*p* < 0.001) [191].

### 3.8. Glycyrrhiza glabra

*Glycyrrhiza glabra*, commonly known as licorice, is one of the most important herbal medicines for traditional Chinese medicine and Japanese Kampo medicine [192]. It is traditionally used to relieve inflammation, gastric and peptic ulcers, arthritis, eye and liver disorders, hyperacidity, and sex-hormone imbalance [193,194,195,196,197,198,199,200,201]. This plant has attracted the attention of the pharmacological field due to its antimicrobial, antiviral, and anti-inflammatory properties [202,203,204,205]. The roots of *G. glabra* have been found to possess many secondary metabolites, with numerous pharmacological properties that contribute to their medicinal use, including flavonoids (such as liquirtin, rhamnoliquirilin, liquiritigenin, and prenyllicoflavone A) and volatile components (including pentanol, hexanol, tetramethyl pyrazine, linalool, and terpinen-4-ol) [206]. The essential oil extracted from the roots of *G. glabra* contains propionic acid, 1-methyl-2-formylpyrrole, benzoic acid, 2,3-butanediol, and ethyl linoleate, among other compounds. The roots of *G. glabra* are also composed of 20% moisture, 3–16% sugars, 30% starch, and 6% ash [207].

The main biologically active components of *G. glabra* are dipotassium glycyrrhizinate, glycyrrhizin, also known as glycyrrhizic acid, and its aglycone, glycyrrhetinic acid [206]. Dipotassium glycyrrhizinate has similar properties to those of corticosteroids, namely, anti-inflammatory, antiallergic, and antibiotic activities, without the side effects of allergic reactions on the skin [208]. This property is due to dipotassium glycyrrhizinate’s ability to efficiently inhibit the activity of phospholipase A2 enzyme, which is necessary for several inflammatory processes [209,210,211]. Moreover, it is able to avoid damage to the extracellular matrix by inhibiting the activity of hyaluronidase enzyme, histamine release, inflammatory chemical mediators, leukotrienes, and prostaglandins [212]. Glycyrrhizic acid inhibits prostaglandin E2 synthesis by suppressing the activity of COX-2, resulting in the augmentation of NO production through the enhancement of iNOS mRNA secretion and indirectly preventing platelet aggregation [211,213,214]. The anti-inflammatory activity of glycyrrhizic and glycyrrhetinic acids is realized through cytokines such as 1β, IL-4, IL-5, IL-6, IL-8, IL-10, IL-12, and IL-17, IFN-γ, and TNF-α [215,216,217]. Moreover, these compounds also present immunomodulatory activity through their interaction with different transcription factors, such as NF-κB, as well as signal transducers and activator of transcriptions (STAT- 3 and STAT-6) [215].

Najafi et al. conducted a double-blind clinical trial to evaluate the potential effect of *G. glabra* extract on cancer patients under head and neck radiotherapy [207]. The experimental group received a 50% extract of *Glycyrrhiza* (hydroalcoholic extract) and the placebo group received a brown-colored water. The patients were asked to use 20 cc twice per day for 14 days after the beginning of RT. According to the results obtained, *Glycyrrhiza* extract efficiently decreased the OM, wound size, and irritation (*p* < 0.001) [207]. The effect of *G. glabra* on head and neck cancer patients receiving RT was also evaluated in a small randomized study with six patients who were assigned to receive a licorice mucoadhesive film or a placebo mucoadhesive film [218]. The level of pain and the mucositis severity were significantly lower in the licorice-mucoadhesive-film-receiving patients in the last 2 weeks of the clinical trial (weeks 3 and 4) (*p* < 0.05) [218].

The efficiency of a *G. glabra* root extract in preventing CT-induced OM in colon cancer patients was evaluated in a double-blind randomized clinical trial that involved 72 patients [219]. The treatment group received 5% licorice root extract, and the control group received a combined mouthwash composed of aluminum, magnesium, diphenhydramine, nystatin powder, and 2% lidocaine. For one week, from the first day of CT, both mouthwashes were used daily, every 8 h, at a dose of 10 cc. The researchers did not observe differences between the two groups in terms of the incidence and severity of OM (*p* > 0.05) [219].

### 3.9. Matricaria Recutita

The chamomile plant, *Chamomilla recutita* or *Matricaria recutita*, one of the most common medicinal plants, is characterized by flowers with anti-inflammatory, antibacterial, and antifungal properties [220]. It is mainly used to treat different inflammatory conditions of the skin and mucosa, as it promotes faster a wound-healing process in comparison to corticosteroids [220,221]. *M. recutita* owes its therapeutic activity to chamazulene, α-bisabolol, bisabolol oxides, spiroethers, and flavonoids [220]. Flavonoids—in particular, apigenin-7-glucoside—have been found to be responsible for the anti-inflammatory activity that may be involved in recuperation from OM [222]. Pre-clinical studies showed evidence of the anti-inflammatory action of *M. recutita* through the inhibition of COX-2 and IL-6 production [223,224].

In a small comparative study with random assignment, dos Reis et al. evaluated the efficacy of *M. recutita* infusion cryotherapy for the prevention and reduction of the intensity of OM in gastric and colorectal cancer patients [225]. The study was performed during the first course (5 days) of CT. The patients in the *M. recutita* group received a cup of ice chips made with an *M. recutita* infusion at 2.5%, while the control group received a cup of ice chips made with pure water. The patients in both groups were instructed to swish the ice chips around in their mouths for at least 30 min, starting 5 min before the CT infusion. The *M. recutita* group presented less pain and had no ulcerations when compared to the control group [225]. The effects and the percentage of extract necessary to reduce the incidence and intensity of OM in patients undergoing hematopoietic stem cell transplantation were assessed in a randomized, controlled, phase II clinical trial [221]. All 40 patients received standard oral care, while the treatment group received an additional mouthwash containing a liquid extract of *M. recutita* at 0.5%, 1%, or 2%. When compared with the control group, the *M. recutita* group at 1% (equivalent to 0.108 mg of apigenin-7-glucoside/mL) demonstrated have reduced incidence, intensity, and duration of OM in patients undergoing hematopoietic stem cell transplantation (*p* < 0.01) [221]. Shabanloei et al. performed a randomized, double-blind clinical trial between alloporinol and *M. recutita* extract in the prevention of OM in CT-receiving patients [226]. Group 1 received 5 mg/mL of allopurinol, group 2 received a solution of 8 g of *M. recutita* in 50 cc, and the control group received a normal saline solution as a mouthwash. All patients gargled daily, four times per day, for the 16 days following the beginning of CT. The researchers concluded that both the allopurinol and *M. recutita* mouthwashes were effective in reducing post-CT OM, with no significant differences in the mean stomatitis (*p* = 0.59) and stomatitis pain (*p* = 0.071) [226].

### 3.10. Calendula officinalis

*Calendula officinalis*, commonly known as marigold, has been used for centuries as a topical and oral herbal remedy due to its bactericidal, antioxidant, anti-inflammatory, antiseptic, hepatoprotective, and anti-metastatic effects, with applications in blood purification and treatment of herpes, keratolytic radiation dermatitis, wounds, and scars, and as an antispasmodic [227,228,229]. The main compounds that contribute to its medicinal use are triterpenoids, flavonoids, oleanolic acid, faradiol, glycosides, quinones, tannins, coumarins, carotenoids, saponins, alkaloids, phenolic acids, and amino acids [227]. Triterpenoids provide anti-inflammatory and anti-edematous effects, in addition to stimulating the proliferation of fibroblasts, possibly through the inhibition of COX-2, C3-convertase, and 5-lipoxygenase [230,231,232]. Flavonoids are reported to have anti-inflammatory, antioxidant, and anti-edematous properties, in addition to their inhibition of lipoxygenase enzymes and mast cells [233].

The potential of *C. officinalis* extract for the healing of 5-fluorouracil-induced OM was studied in hamsters [229]. OM was induced in 60 male hamsters on days 0, 5, and 10 through the intraperitoneal administration of 5-fluorouracil (60 mg/kg). The cheek pouch was scratched with a needle once per day, from day 1 until day 12, when erythematous changes were noted. The treatment of OM started on days 12–17 with the topical application of a gel once a day. The animals were divided into four groups: 12 without treatment as control animals, 15 treated with 5% *C. officinalis* gel, 15 treated with 10% *C. officinalis* gel, and 15 treated with the gel base. The *C. officinalis* gel (5% and 10%) significantly reduced the microscopic and macroscopic scores of OM when compared with the gel base and the control group. Moreover, the animals of the treatment groups gained more weight than those in the gel base and the control groups [229]. In humans, the effect of *C. officinalis* on OM was evaluated in a placebo-controlled clinical trial with 40 patients with neck and head cancers under RT or concurrent CT [234]. Patients were given 5 mL of either placebo or a 2% *C. officinalis* extract gel mouthwash to be held for at least 1 min in the oral cavity two times per day. Compared to the placebo group, the intensity of OM was significantly lower in the *C. officinalis* mouthwash group at weeks 2, 3, and 6 (*p* < 0.048). According to the same study, the high content of flavonoids and phenolic compounds and the antioxidant activity may be responsible for the protective effect of *C. officinalis* in RT-induced OM [234].

### 3.11. Other Compounds

In addition to the compounds mentioned above, different experiments were also performed to evaluate the potential of other natural compounds for preventing/treating OM. However, due to the low number of studies published, not only regarding the OM application, but also with respect to the molecular mechanism of action enrolled, a section was not dedicated to them in this review. Table 4 summarizes the different natural products in these circumstances.

## 4. Conclusions and Future Perspectives

OM is a common and incapacitating side effect of antineoplastic therapies. The increased knowledge of its pathogenesis allows a better prediction of a patient’s risk with the aim of adapting the management protocols and improving the development of new therapies. Nevertheless, standard guidelines for preventing and treating OM do not display significant effectiveness. The interest in natural products as potential therapeutic drugs has increased in recent years, as they have the advantage of being accessible and generating minimal side effects, with potential properties that include anti-inflammatory, antioxidant, antimicrobial, antiulcerative, and wound-healing capacities. In addition, over recent years, there have been multiple efforts to develop naturally based therapies, with natural compounds being tested in model organisms and clinical trials that are currently ongoing. However, the environment of the oral cavity is a complex system that is divided into two functional layers—the epithelium (thick and avascular) and the underlying tissue (vascular)—that are anatomically different, which affects their permeability to drugs and the capacity for maintaining a system for a certain period [255]. The buccal mucosa, which is composed of epithelial cells, provides a large surface area of almost 100 cm^2^ [256]. This area is ideal for attaching a drug delivery system, providing a permeability that is 4 to 4000 times higher than that of skin [256,257,258,259]. Oral administration provides the advantage of a simple administration that does not suffer from the first-pass metabolism and that is safe and increases the drug availability. In addition, this route has a rapid action, reduced side effects, easy access to the local condition, and great patient compliance [255,260]. These characteristics make the buccal mucosa an optimal solution for the systemic and local treatment of OM [261]. However, it also has limitations that are associated with a functionalized protective barrier. The presence of saliva and its enzymatic action, as well as the constant mechanical pressure caused by eating and speaking movements, may compromise the penetration of the drug present in the delivery system; as such, the application of mucoadhesive components may be required to solve this issue, but this can compromise the therapeutic effectiveness [255,257,258,259].

Due to the characteristics of the oral cavity, it is necessary to develop novel strategies for overcoming topical delivery, such as mucoadhesive dosage forms (e.g., films, tablets). For the treatment of oral diseases, the most suitable formulations investigated were in the form of tablets, films, sprays, mouthwashes, gels, and pastes [24,259,262]. Gel and film formulations were evaluated in hamsters with CT-induced mucositis. By the 28th day, the hamsters’ mucosa appeared to be healed, as no erythema or edema was visible. These results proved their efficiency, as the animals’ survival was higher than in the control group, and these treatments showed promising potential for a function as an occlusive patch and for delivering therapeutic compounds [261]. Films containing ethanolic propolis extract also presented optimal mucoadhesion capacity, ensuring the release of propolis compounds, a good stability, a high swelling capacity, and antimicrobial effects against *S. aureus* [263]. In addition, the incorporation of nanoparticles in the forms of dosage for buccal drug delivery has recently been encouraged [24,256,264]. Furthermore, nanoparticles could transport many therapeutic agents [24,256]. Functional and biocompatible carriers that display chemical stability are sought in the innovation of buccal drug delivery systems [264,265,266,267]. Chitosan is an example of a biopolymer that is biologically safe and bioadhesive, and it has been used in several studies for the development of drug delivery systems, as it has longer retention periods in the oral mucosa [261,268,269]. In addition, it inhibits the attachment of *C. albicans* to human oral mucosal cells [261,268,269].

**Figure 2:**
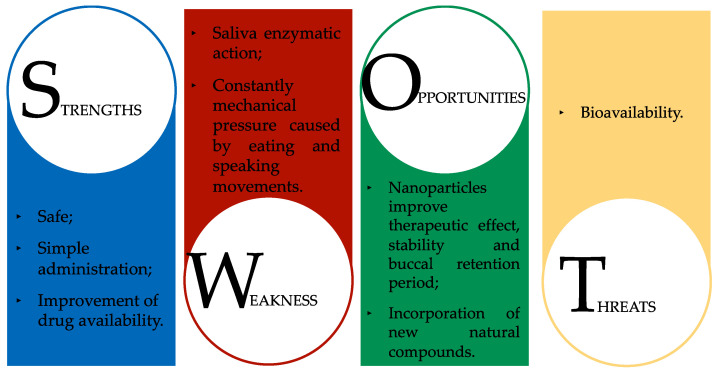
SWOT analysis for the possible use of natural products to prevent/treat OM.

A SWOT diagram (Figure 2) was constructed with the aim of summarizing the previously described strengths, weaknesses, opportunities, and threats of employing natural products for the prevention/treatment of OM.

Despite the significant advances made in this area, more investigations are needed to ensure that these formulations reach the pharmaceutical market, and few have been published regarding this topic with natural products.

## Figures and Tables

**Table 1:** Available clinical scales for oral mucositis assessment. Adapted from [16]. NA—Not applicable.

Scale	Grade 0	Grade 1 (Mild)	Grade 2 (Moderate)	Grade 3 (Severe)	Grade 4 (Life-Threatening)	Grade 5 (Death)
WHO	No findings	Oral erythema and soreness; no ulcers	Oral erythema, ulcers; solid diet tolerated	Oral ulcers; liquid diet only	Oral alimentation impossible	NA
CTCAE	None	Asymptomatic or mild symptoms; intervention not indicated	Moderate pain or ulcer that does not interfere with oral intake; modified diet indicated	Severe pain, interfering with oral intake	Life-threatening consequences; urgent intervention indicated	Death
RTOG	No change over baseline	Irritation; may experience mild pain, not requiring analgesics	Patchy mucositis that may produce an inflammatory serosanguinous discharge; may experience moderate pain requiring analgesia	Confluent, fibrinous mucositis; may include severe pain requiring narcotics	Ulceration, hemorrhage, or necrosis	NA

**Table 2:** Risk factors related to patients, tumors, and treatments in the development of oral mucositis.

Risk Factor	Criteria	References
Related to patient		
Age	Extremities	[9,12,25,45,56,57,58]
Gender	Female	[2,3,10,23]
Body mass index (BMI)	Low and high body mass index	[2,3,10,23]
Dental prosthesis	Orthodontics and prosthesis	[9,11]
Education	Lack of health literacy	[7,55,59,60]
Oral hygiene	Oral hygiene less than 2 times/day Periodontal disease	[2,3,10,23]
Comorbidities	Diabetes mellitus, renal and hepatic dysfunction	[16,54,61]
Leucocytes	Neutropenic patients are immunocompromised	[2,18,45]
Alcohol	Use of alcohol prior to and during treatment	[2,3,18,45]
Smoking	Smoking prior to and during treatment increases the severity	[5,45]
Genetics	Genetic polymorphisms (e.g., TNF-α)	[10,45]
Mucosal trauma	Sharpened teeth	[5]
Related to tumor		
Types of cancer	Solid tumors have higher risk, mainly those located near oral cavity	[12,45,47]
Related to treatment		
Type of treatment	5-fluorouracil, Doxorubicin, Methotrexate, Cisplatin, Vinblastine, Mitomycin, Transtuzumabe, Docetaxel, Melphalan	[10,16,54]
Dose	High doses over short periods and their extension	[10,16,54]
Type of administration	Intravenous	[2,10,16,45,54]
Microbiota		

**Table 3:** Management of Oral Mucositis Guidelines created by the Multinational Association for Supportive Care in Cancer and the International Society of Oral Oncology.

Intervention	Aim	MASCC/ISOO Guideline Category	Results	References
Oral care	Prevention	Suggestion	Increases patient’s awareness and enhances their compliance with treatment	[5,37,46,60,63,67,68,69]
Oral cryotherapy	Prevention	Recommendation	Local vasoconstriction that minimizes drug absorption	[11,46,70,71,72,73]
Photobiomodulation therapy	Prevention	Recommendation	Promotes wound healing and has an anti-inflammatory effect	[23,26,39,40,41,46]
Benzydamine mouthwash	Prevention	Recommendation	Anti-inflammatory properties by inhibiting the production of pro-inflammatory cytokines	[46,53,54,74,75,76]
Keratinocyte growth factor-1 (palifermin)	Prevention	Recommendation	Proliferation and restoration of epithelial cells	[26,27,46]
Glutamine	Prevention	Suggestion	It is used by cells of the immune system	[28,30,34,46]
Honey	Prevention	Suggestion	Inhibits bacterial growth and enhances healing rate	[32,35,37,38,46,77]
Patient-controlled analgesia (e.g., 0.2% morphine mouthwash)	Treatment	Recommendation	Pain management	[46,78,79]
Zinc supplements	Prevention	Suggestion	Prevents lipids peroxidation and replaces redox-reactive metals	[28,30,46]
Doxepin mouthwash	Treatment	Suggestion	In topical application, it has analgesic and anesthetic properties	[46,78,80]
Vitamin E	Prevention	Suggestion	Antioxidant that may protect tissue damage from free oxygen radicals	[28,30,31,46,73]
Amifostine	Prevention	Suggestion	Reduces DNA strand breaks, recruits ROS scavengers, and preserves salivary glands, endothelium, and connective tissue integrity	[4,33,46]

**Table 4:** Summary of studies with natural products for prevention/treatment of oral mucositis.

Name	Properties/Mechanisms	Application	Experimental Setting/Model	References
Manuka (*Leptospermum scoparium*) essential oil	Anti-inflammatory, analgesic, antimycotic, and antibacterial	Mouthwash	Randomized placebo-controlled trial	[235]
Kanuka (*Kunzea ericoides*) essential oil	Anti-inflammatory, analgesic, antimycotic, and antibacterial	Mouthwash	Randomized placebo-controlled trial	[235]
Indigo root (*Isatis indigotica*)	Anti-inflammatory and antiviral	Mouthwash	Randomized clinical trial	[236]
*Rhodiola algida*	Immunomodulatory effects	Mouthwash	Randomized clinical trial	[237]
*Thymus spp. L*	Antiseptic, anti-inflammatory, antimicrobial, and antimycotic	Mouthwash	Randomized pilot study	[238]
*Eucalyptus*	Antibacterial, antiviral, antifungal, anti-inflammatory, analgesic, and antioxidant	Topical gel	Hamsters	[239]
*Zizyphus jujuba*	Anti-inflammatory, analgesic, and wound healing	Topical gel and dietary	Hamsters	[240]
*Zataria multiflora*	Carminative, stimulant, diaphoretic, diuretic, antiseptic, anesthetic, antispasmodic, anti-hermitic, antidiarrheal, and analgesic	Mouthwash	Randomized clinical trial	[241]
*Carapa guianensis* oil	Anti-inflammatory, analgesic, and antimicrobial	Topical gel/swab	Controlled and randomized clinical trial/ hamsters	[242,243]
*Plantago ovata*	Antioxidant, anti-inflammatory, and antibacterial	Mouthwash	Randomized cross-over clinical trial	[244]
*Achillea millefolium*	Antimicrobial and anti-inflammatory	Mouthwash	Double-blind, randomized, controlled trial	[82]
*Vaccinium myrtillus*	Antioxidant, cardioprotective, neuroprotective, anti-inflammatory, and anticarcinogenic	Topical application, gavage administration, mouthwash	Clinical trials, Hamsters	[245,246,247]
*Carum carvi*	Antioxidant, antidiabetic, antifungal, and antimicrobial	Topical gel	Hamsters	[248]
*Pistacia atlantica*	Antioxidant and anti-inflammatory	Topical gel	Hamsters	[249,250]
*Hypericum perforatum*	Antioxidant and anti-inflammatory	Topical gel	Hamsters	[251]
*Elaeagnus angustifolia*	Anti-inflammatory, analgesic, and wound healing	Topical gel	Hamster	[252]
*Trachyspermum ammi*	Anti-inflammatory, antiviral, antifungal, antioxidant, and analgesic	Topical gel	Hamsters	[250]
*Hippophae rhamnoides*	Antioxidant, anti-inflammatory, antimicrobial, and anti-ulcerogenic	Gavage administration	Rats	[253,254]

## Data Availability

Data are available on request due to restrictions, e.g., privacy or ethical restrictions.
